# Longitudinal assessment of racial disparities in juvenile idiopathic arthritis disease activity in a treat-to-target intervention

**DOI:** 10.1186/s12969-020-00485-y

**Published:** 2020-11-13

**Authors:** Joyce C. Chang, Rui Xiao, Jon M. Burnham, Pamela F. Weiss

**Affiliations:** 1 Division of Rheumatology, Children’s Hospital of Philadelphia, Philadelphia, PA 19104 USA; 2 Center for Pediatric Clinical Effectiveness, Children’s Hospital of Philadelphia Research Institute, 2716 South St, 11th Floor, Philadelphia, PA 19146 USA; 3 Department of Pediatrics, University of Pennsylvania Perelman School of Medicine, Philadelphia, PA 19104 USA; 4 Department of Biostatistics, Epidemiology and Informatics, University of Pennsylvania Perelman School of Medicine, Philadelphia, PA 19104 USA; 5 Office of Clinical Quality Improvement, Children’s Hospital of Philadelphia, Philadelphia, PA 19104 USA; 6 Center for Pharmacoepidemiology Research and Training, University of Pennsylvania, Philadelphia, PA 19146 USA

**Keywords:** Juvenile arthritis, Healthcare disparities, Patient outcome assessment, Patient reported outcome measures, Pediatrics

## Abstract

**Background:**

We sought to evaluate racial disparities in disease outcomes among children with polyarticular juvenile idiopathic arthritis (JIA) during a treat-to-target (TTT) intervention with clinical decision support (CDS).

**Methods:**

This was a retrospective analysis of a TTT-CDS strategy integrated into clinical practice for children with polyarticular JIA at a single center from 2016 to 2019. The primary outcome was the clinical Juvenile Arthritis Disease Activity Score (cJADAS-10). We used multivariable linear regression to assess racial differences in disease outcomes at the index visit (first visit after implementation). The effect of race on disease outcomes over time was estimated using linear mixed-effects models, stratified by incident or prevalent disease.

**Results:**

We included 159 children with polyarticular JIA, of which 74, 13 and 13% were white, black, and Asian/other, respectively. cJADAS-10 improved significantly over time for all race categories, while the rates of improvement did not differ by race in incident (*p* = 0.53) or prevalent cases (*p* = 0.58). cJADAS-10 over time remained higher among black children compared to white children (β 2.5, *p* < 0.01 and β 1.2, *p* = 0.08 for incident and prevalent cases, respectively). Provider attestation to CDS use at ≥50% of encounters was associated with a 3.9 greater reduction in cJADAS-10 among black children compared to white children (*p* = 0.02).

**Conclusion:**

Despite similar rates of improvement over time by race, disparities in JIA outcomes persisted throughout implementation of a TTT-CDS approach. More consistent CDS use may have a greater benefit among black children and needs to be explored further.

## Background

Juvenile idiopathic arthritis (JIA) is an umbrella term for a heterogeneous group of chronic childhood inflammatory arthritides that may lead to joint damage, impaired growth, and impaired physical function if left under-treated. JIA is categorized by the International League Against Rheumatism (ILAR) classification system into seven different categories. Two categories, rheumatoid factor (RF) positive and RF negative polyarticular JIA, affect 5 or more joints at onset of disease. Children with polyarticular JIA are more likely than those with other categories of JIA to experience prolonged periods of active disease [1]. Therapeutic advances in recent decades have greatly improved outcomes of children with polyarticular JIA, however there remains great variability in the timing and use of disease-modifying antirheumatic drugs (DMARDs) and biologic therapies [2]. Furthermore, racial disparities in treatment outcomes for polyarticular JIA are not well characterized.

In a cross-sectional study using the Childhood Arthritis and Rheumatology Research Alliance registry, African American children with polyarticular JIA had a nearly two-fold higher odds of joint damage compared to their white counterparts [3]. Similarly, a wide variety of factors have been hypothesized to contribute to worse outcomes among African American adults with rheumatoid arthritis, including genetic factors, decreased access to care, lower rates of DMARD and biologic use, other socioeconomic determinants of health, and implicit bias in health care delivery [4–9]. Quality improvement initiatives that lead to better delivery of care processes and more productive interactions between health care providers and patients may help reduce racial disparities [10].

Treat-to-target (TTT) is one method of improving the delivery of care processes that involves systematic measurement of disease activity using standardized outcome measures and adjusting treatment accordingly. TTT approaches have been shown to improve outcomes in adults with rheumatoid arthritis [11, 12]. Similarly, there is more recent evidence to suggest that TTT strategies result in better disease control in children with JIA compared to unstructured treatment [13, 14]. Initial recommendations for the adoption of TTT principles in JIA were put forth by an international task force [15, 16]. We have previously described the implementation of a TTT approach for polyarticular JIA in a real-world clinical setting with standardized, real-time outcome measurement augmented by clinical decision support (CDS) [17]. The key components reflected TTT guidelines for rheumatoid arthritis (later recapitulated in the recommendations for JIA) [18], and included aiming for clinical remission, obtaining validated composite disease activity measures at the point of care, adjusting drug therapy to achieve the treatment target, and communicating these concepts with families. The novel component was the development of a CDS tool to support standardization of how drug therapy was adjusted at the point of care. While the initiative resulted in broad population-level improvements in disease activity, it is not known whether there were pre-existing racial disparities in disease activity outcomes, or if there were racial differences in the subsequent response to the intervention.

The main objectives of this study were to: 1) determine whether black children with polyarticular JIA had worse disease outcomes relative to white children at the start of the TTT-CDS intervention, and 2) determine whether changes in disease activity outcomes over time differ by race within the context of implementation of the intervention. Our hypothesis is that there are pre-existing racial disparities in JIA disease activity that can be improved upon by integrating standardized outcome measurement and CDS into routine clinical practice.

## Methods

### Treat-to-target intervention

To improve outcomes among children with polyarticular JIA, a quality improvement effort detailed previously was undertaken at the Children’s Hospital of Philadelphia (CHOP) in 2016 [17]. The interventions consisted of standardized disease activity measurement, review of disease activity at the point of care, and decision support to reduce treatment variability. In February 2016, we launched a standardized outcome assessment method using a Research Data Capture (REDCap) survey tool to capture physician and patient-reported components of the clinical Juvenile Arthritis Disease Activity Score (cJADAS-10) [19], pain scores, and Patient Reported Outcomes Measurement Information System (PROMIS®) mobility and upper extremity function T-scores [20], which have previously been validated for JIA [21]. The REDCap report integrated these data into an automated measure of disease activity at the point of care. Providers then performed a target attestation in the electronic health record to record whether disease activity, physical function and pain scores were at target. A new after visit summary was designed to automatically populate the target assessment to review with patients and caregivers at the point of care.

### Clinical decision support

In April 2016, we augmented the TTT intervention by implementing decision support algorithms for polyarticular JIA, which were iteratively designed to standardize medication selection and dosing, as well as treatment duration. Branching logic in the REDCap tool was used to show providers the CDS algorithm relevant to their patient at the point of care, as previously described [17]. Providers were asked to complete an attestation in the electronic health record to whether or not they utilized the CDS algorithm at each encounter.

### Data source and study population

The source population for this study was children with JIA evaluated in the rheumatology clinic at CHOP from February 2016 through December 2019. To be included in this analysis, patients had to fulfill criteria for the ILAR categories of RF positive or RF negative polyarticular JIA, and have at least 2 visits at least 1 month apart following implementation of the TTT-CDS intervention. The first visit following implementation of CDS was considered the index date for each patient. Therefore, patients newly diagnosed with JIA after the implementation date were exposed to the intervention starting from the time of diagnosis, while those with prevalent disease were first exposed during follow-up after the implementation date. Data for each was extracted from the Qlikview Platform, an automated data visualization tool that integrates the REDCap survey data for each JIA visit. ILAR category and medication use were confirmed by manual chart review. An exemption was approved for this study by the Children’s Hospital of Philadelphia (CHOP) Institutional Review Board (#18–014808) for the conduct of secondary research for which consent is not required.

### Study measures

The primary outcome was disease activity, as defined by the three-variable cJADAS-10. The cJADAS-10 is a validated measure of disease activity for JIA and is calculated from the active joint count, physician global assessment, and parent/patient global assessment, which were measured routinely at every visit as part of the standardized outcome assessment intervention. Scores range from 0 to 30, with scores of ≤1.5, 2.5–8.5, and > 8.5 corresponding to low, moderate and high disease activity, respectively [22]. As secondary outcomes, we also longitudinally assessed individual cJADAS-10 components, patient-reported pain, PROMIS® mobility, and PROMIS® upper extremity function over time. Time in months following the index visit was the primary exposure for all longitudinal models, and self-reported race was the covariate of interest, categorized as 1) black/African American, 2) white, and 3) Asian or other race.

*Additional time-independent covariates* included age at the index visit, sex, self-reported ethnicity, commercial versus public insurance, disease duration, baseline disease activity score, and CDS use. Disease duration was categorized by incident cases (≤ 6 weeks since initial JIA diagnosis) and prevalent cases (> 6 weeks since diagnosis). CDS use at the subject-level was determined by whether the provider attested to using the CDS algorithms in ≥50% of encounters. *Time-varying covariates* included non-biologic DMARD use and biologic use.

### Statistical analysis

Baseline demographic factors and disease activity were summarized using standard descriptive statistics and compared between race categories using Fisher’s exact tests for categorical variables and Kruskal-Wallis tests for continuous variables.

#### Cross-sectional analysis of racial differences in disease outcomes at the index visit

We performed pairwise comparisons of cJADAS-10 at the index visit by race using Wilcoxon rank sum tests. To estimate the independent effect of race on baseline cJADAS-10, we performed multivariable linear regression with forward stepwise selection methods (*p*-value ≤0.2 for entry, *p*-value > 0.3 for removal).

#### Longitudinal analysis of racial differences in disease outcomes over time

Linear mixed-effects models were used to estimate the effect of race on cJADAS-10 over time during the TTT-CDS intervention, stratified by incident versus prevalent cases. This model accounts for within-subject correlation due to repeated measures by including subject-specific random effects, which were modeled by a Markov covariance matrix to account for unequally spaced and unbalanced data. We assumed random slope in the mixed-effects model to estimate subject-specific rates of change in cJADAS-10 over time, and random intercept to allow for variation of subject-specific cJADAS-10 scores at time zero. LOWESS plots were used to visually assess linearity between the outcome and the covariates. Time-squared was included in the model to capture potential non-linear trends. All models were adjusted for baseline cJADAS-10, and stepwise forward selection methods were used to select additional covariates for inclusion. RF positivity, medication use and insurance status were determined a priori as potential confounders and were forced into the model and retained if the coefficient of interest changed by more than 15%. To determine whether change in disease activity over time differed by race, we tested interactions between race and time.

We performed several secondary analyses. First, separate linear mixed-effects models were used to test for racial differences in each of the secondary outcomes over time. Secondly, to determine whether racial differences were modified by use of the CDS algorithms, we also identified JIA cases in which the provider attested to using CDS in at least 50% of encounters and tested interactions between race and CDS use. Lastly, we used mixed-effects logistic regression models to determine whether changes in biologic use over time differed by race. All statistical analyses were conducted in Stata, version 14.2 (Stata Corporation, College Station, TX) using a pre-determined significance level of 0.05.

## Results

There were 159 children with polyarticular JIA with a median of 6 visits per patient following TTT-CDS implementation (interquartile range [IQR] 4–8), totaling 998 visits. Median follow-up time was 30 months (IQR 20–36). Incident diagnoses comprised 36% of cases. The median age at the index visit was 12 years (IQR 7.2–14.7). The majority of children were white (74%) and 13% were black. Demographic characteristics and baseline disease outcome measures by racial category are shown in Table 1. A higher proportion of black children were male, RF positive, and publicly insured compared to their white counterparts (Table 1).

**Table 1:** Demographic and disease characteristics by race at index visit

	White	Black	Asian/Other	
	*N* = 117	*N* = 21	*N* = 21	*p*-value*
Age, median (IQR)	11.6	13.0	10.6	0.20
	(7.2–14.6)	(9.3–16.0)	(5.9–14.3)	
Male sex, n (%)	14 (12%)	6 (29%)	7 (33%)	0.02
Hispanic ethnicity	3 (3%)	0 (0%)	4 (19%)	0.01
Public insurance	47 (40%)	15 (71%)	12 (57%)	0.02
Incident diagnosis (< 6 months)	37 (32%)	12 (57%)	9 (43%)	0.08
Rheumatoid factor positive	13 (11%)	11 (52%)	5 (24%)	< 0.01
Non-biologic DMARD use	61 (52%)	12 (57%)	13 (62%)	0.71
Biologic use	48 (41%)	6 (29%)	6 (29%)	0.43
cJADAS-10, median (IQR)	5.5	13.6	6.2	0.02
	(0.5–13.6)	(9.5–19.0)	(0.7–17.0)	
Physician global assessment	1.0	4.0	1.5	< 0.01
	(0.0–3.0)	(2.0–5.0)	(0.0–4.5)	
Patient/parent global assessment	2.0	4.4	2.8	0.06
	(0.4–4.0)	(2.1–6.8)	(0.3–4.9)	
Joint count	0.0	5.0	1.0	0.06
	(0.0–6.0)	(1.0–9.0)	(0.0–5.5)	
Pain	1.9	6.3	3.2	0.01
	(0.5–4.8)	(3.1–7.3)	(0.4–4.8)	
PROMIS® upper extremity	49.0	31.8	47.0	0.05
	(34.1–56.7)	(26.1–55.9)	(34.1–56.7)	
PROMIS® mobility	48.0	35.0	48.0	0.02
	(40.0–58.5)	(29.2–51.0)	(32.0–56.0)	

* Fisher’s exact test for categorical variables, Kruskal-Wallis test for continuous variables

*IQR* interquartile range; *DMARD* disease-modifying anti-rheumatic drug; *cJADAS-10* three-variable clinical Juvenile Arthritis Disease Activity Score; *PROMIS®* Patient Reported Outcomes Measurement Information System

### Racial differences in disease outcomes at the index visit

The median cJADAS-10 at the index visit was significantly higher for black children (median 13.6 [IQR 9.5–19.0]) compared to their white counterparts (2.4 [IQR 0.1–6.8], *p* < 0.01), and compared to all non-black race categories combined (5.6 [IQR 0.5–13.8], *p* < 0.01). After adjustment for age, disease duration, and DMARD use, black race was associated with a 3.0 point higher cJADAS-10 at the index visit compared to white race (95% CI [0.5, 5.5], *p* = 0.02).

Of the three cJADAS-10 components, physician global and patient global scores at the index visit were significantly higher in black children compared to their white peers (adjusted β 1.1, 95% CI [0.2, 2.1], *p* = 0.02 and β 1.4, 95% CI [0.2, 2.5], *p* = 0.02, respectively). The estimated difference in joint count was not statistically significant (β1.5, 95% CI [− 0.8, 3.8], *p* = 0.19). Black children also had significantly higher adjusted pain scores (β 1.8, 95% CI [0.8, 2.9], *p* < 0.01) and lower mobility at the index visit (β − 6.4, 95% CI [− 11.1, − 1.8], *p* = 0.01). There was no significant difference in upper extremity physical function (β − 4.1, 95% CI [− 9.7, 1.6], *p* = 0.16).

### Racial differences in disease outcomes over time

**Fig. 1:**
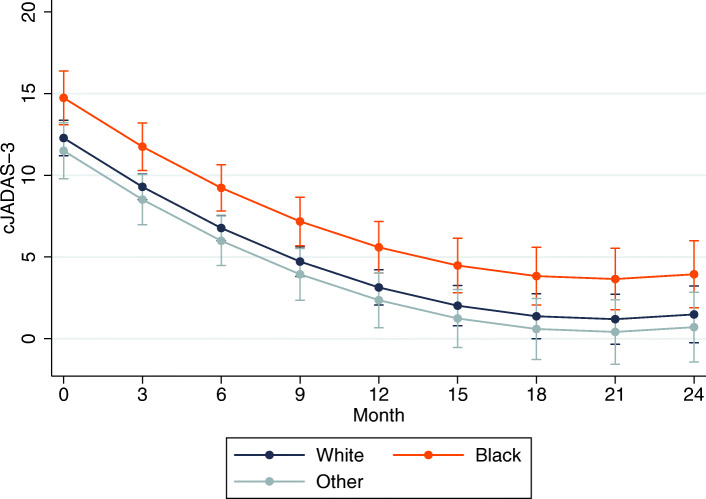
Predicted margins of disease activity over time by race in incident JIA. Predicted mean disease activity scores over time among incident polyarticular JIA cases by race, with 95% confidence intervals from the linear mixed-effects regression model, at means of all other covariates (age, baseline cJADAS score, RF positivity, non-biologic DMARD use, biologic use)

**Fig. 2:**
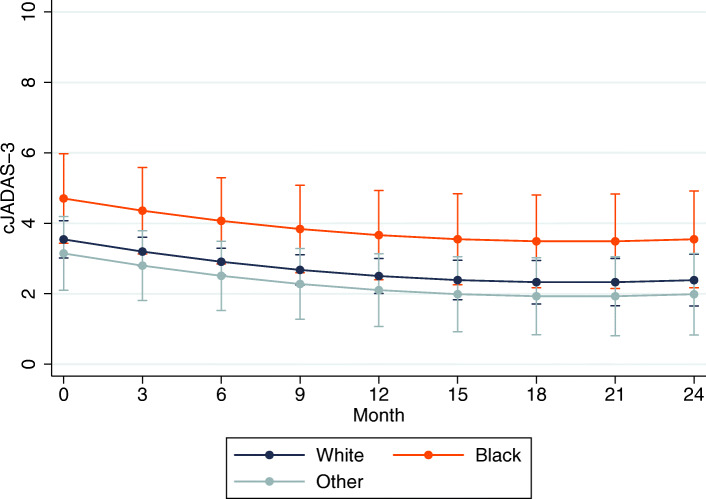
Predicted margins of disease activity over time by race in prevalent JIA. Predicted mean disease activity scores over time among prevalent polyarticular JIA cases by race, with 95% confidence intervals from the linear mixed-effects regression model, at means of all other covariates (baseline cJADAS score, insurance status, biologic use)

On average, there was a statistically significant improvement in cJADAS-10 scores over time across all race categories for both incident (Table 2) and prevalent cases (Table 3). Among incident cases (*n* = 58), black children (*n* = 12) had on average a 2.5 point higher adjusted cJADAS-10 over time compared to their white counterparts (95% CI [0.8–4.1], *p* < 0.01) (Table 2). The predicted marginal mean cJADAS-10 was 5.6 for black children versus 3.1 for white children at month 12 and 3.9 vs. 1.5 at month 24 (Fig. 1). There were no significant differences between Asian/other race and white race. Among prevalent cases (*n* = 101), black children (*n* = 9) had on average a 1.2 point higher cJADAS-10 over time compared to their white counterparts (95% CI [− 0.1, 2.4], *p* = 0.08), albeit not statistically significant. Predicted marginal mean cJADAS-10 for prevalent cases was 3.8 for black children vs. 2.7 for white children at month 12 and 3.6 vs. 2.4 at month 24 (Fig. 2). There were no significant differences in the rates of improvement in disease activity over time by race in either incident cases (*p* = 0.53 for interaction) or prevalent cases (*p* = 0.58 for interaction).

**Table 2:** Factors associated with disease activity over time among incident cases of polyarticular JIA

	Unadjusted			Adjusted		
	β	[95% CI]	*p*-value	β	[95% CI]	*p*-value
Age at index visit	0.17	[0.02, 0.32]	0.02	0.11	[− 0.04, 0.25]	0.14
Male sex	0.27	[−1.71, 2.25]	0.79	–		
Race						
White	–			–		
Black	2.83	[1.14, 4.52]	0.00	2.46	[0.80, 4.11]	< 0.01
Asian/Other	−0.70	[−2.51, 1.12]	0.45	−0.78	[−2.46, 0.9]	0.36
Hispanic ethnicity	− 2.81	[−8.06, 2.44]	0.29	–		
Public insurance	1.06	[−0.45, 2.58]	0.17	–		
RF positivity	1.16	[−0.43, 2.75]	0.15	−0.71	[−2.32, 0.9]	0.39
Baseline cJADAS-10	0.47	[0.34, 0.61]	0.00	0.48	[0.36, 0.61]	< 0.01
Non-biologic DMARD use^a^	−1.72	[−2.86, −0.58]	0.00	−2.66	[−3.78, − 1.55]	< 0.01
Biologic use^a^	− 1.46	[− 2.80, − 0.12]	0.03	−1.88	[− 3.14, − 0.61]	< 0.01
Time (months)	− 1.24	[− 1.41, − 1.06]	0.00	−1.07	[− 1.27, − 0.88]	< 0.01
Time-squared	0.03	[0.02, 0.04]	0.00	0.03	[0.02, 0.03]	< 0.01

Marginal effect of covariates on repeated measures of cJADAS over time, estimated from linear mixed-effects models for incident cases of polyarticular JIA

^a^Time-varying covariate

*RF* rheumatoid factor; *cJADAS-10* clinical Juvenile Arthritis Disease Activity Score; *DMARD* disease-modifying anti-rheumatic drug

**Table 3:** Factors associated with disease activity over time among prevalent cases of polyarticular JIA

	Unadjusted			Adjusted		
	β	[95% CI]	p-value	β	[95% CI]	p-value
Age at index visit	0.04	[− 0.09, 0.18]	0.52	–		
Male sex	0.23	[−1.45, 1.91]	0.79	–		
Race						
White	–			–		
Black	3.16	[1.00, 5.32]	< 0.01	1.16	[−0.12, 2.44]	0.08
Asian/Other	−0.39	[−2.24, 1.46]	0.68	−0.40	[−1.45, 0.65]	0.46
Hispanic ethnicity	−0.99	[−3.54, 1.55]	0.44			
Public insurance	1.00	[−0.27, 2.26]	0.12	0.54	[−0.17, 1.25]	0.14
RF positivity	0.36	[−1.57, 2.29]	0.72			
Baseline cJADAS-10	0.56	[0.49, 0.62]	< 0.01	0.54	[0.47, 0.61]	< 0.01
Non-biologic DMARD use^a^	−0.51	[−1.34, 0.32]	0.23			
Biologic use^a^	−1.13	[−2.07, −0.18]	0.02	−0.43	[− 1.09, 0.23]	0.20
Time (months)	−0.15	[−0.24, − 0.07]	< 0.01	−0.13	[− 0.21, − 0.04]	< 0.01
Time-squared	0.004	[0.001, 0.01]	< 0.01	0.003	[0.001, 0.01]	0.01

Marginal effect of covariates on repeated measures of cJADAS over time, estimated from linear mixed-effects models for prevalent cases of polyarticular JIA

^a^Time-varying covariate

*RF* rheumatoid factor; *cJADAS-10* clinical Juvenile Arthritis Disease Activity Score; *DMARD* disease-modifying anti-rheumatic drug

**Fig. 3:**
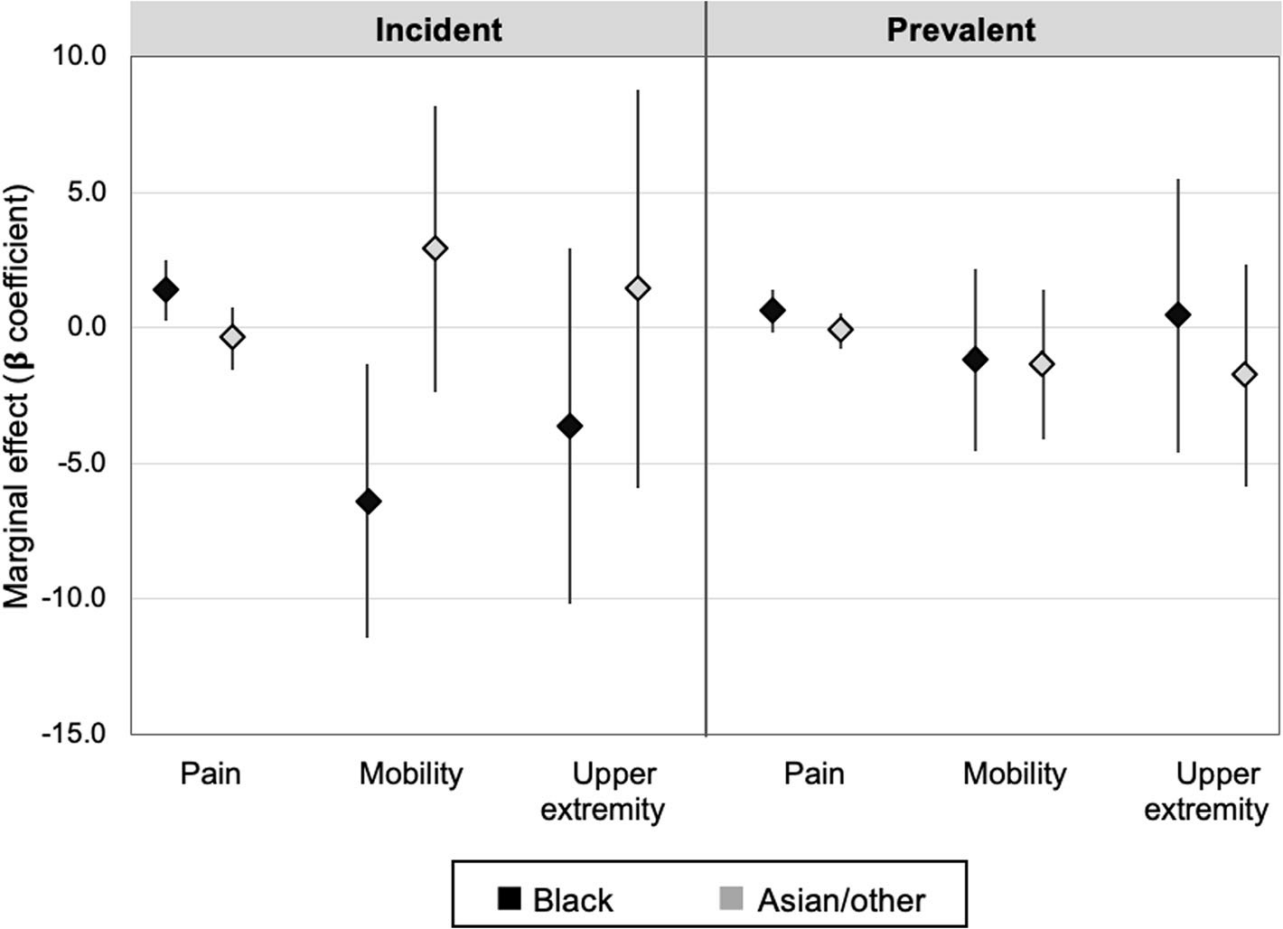
Marginal effects of race on pain and physical function over time. Marginal effects of race on pain, PROMIS® mobility, and PROMIS® upper extremity scores over time among incident and prevalent polyarticular JIA cases. Each beta coefficient is derived from a separate multivariable linear mixed-effects model, representing the mean difference over time in the outcome for each racial category compared to white race (reference group). Bars represent 95% confidence intervals

With respect to individual components of the cJADAS-10, black race among incident cases was associated with worse physician global assessment (β 0.8, 95% CI [0.3, 1.3], *p* < 0.01) and patient global assessment scores over time (β 1.4, 95% CI [0.4, 2.4], *p* = 0.01). Differences in joint count were not statistically significant (β 0.8, 95% CI [− 0.7, 2.3], *p* = 0.32). Black race among prevalent cases was also associated with a higher patient global assessment (β 0.7, 95% CI [0.01, 1.4], *p* = 0.05), but there were no significant differences in physician global (β 0.4, p95% CI [− 0.0, 0.8] *p* = 0.08) or joint count (β − 0.13, 95% CI [− 0.8, 0.5], *p* = 0.70). Of the secondary outcomes assessed, pain and mobility were significantly worse over time among black children with incident disease (Fig. 3). There were no significant racial differences in the rates of change over time in individual cJADAS-10 components, pain or physical function measures.

### Effect of clinical decision support by race

In 74/159 (47%) cases, providers attested to using the CDS tool in at least 50% of encounters. There was no overall association between greater CDS use and disease activity over time in either incident (β − 1.0 (95% CI [− 2.2, 0.3]), *p* = 0.13) or prevalent cases (β − 0.3, 95% CI [− 1.0, 0.4], *p* = 0.43). However, among prevalent cases there was a significant interaction between race and CDS use, in which ≥50% CDS use in black children was associated with an average 3.9 point lower cJADAS-10 over time compared to < 50% CDS use (*p* = 0.02 for interaction).

### Biologic use over time by race

On average, there was an increased odds of biologic use over time (OR 1.1 per month, 95%CI [1.07, 1.14], *p* < 0.01) across the entire cohort. There were no significant differences in the rate of change in biologic use by race (*p* = 0.43 for interaction). However, ≥ 50% CDS use was associated with an increased rate of change in biologic use over time compared to < 50% CDS use (OR 1.1, 95% CI [1.03, 1.16], *p* < 0.01 for interaction).

## Discussion

There are several important findings from this longitudinal study of disease activity in children with polyarticular JIA at an academic center in the US. In the setting of a TTT intervention with standardized outcome assessment and CDS for escalation of therapy, most children with polyarticular JIA had a significant decrease in disease activity, irrespective of race or disease duration. At the start of the intervention, we observed clinically significant pre-existing racial disparities in JIA outcomes. Although rates of improvement in disease activity over time were similar between racial groups, black children had consistently higher disease activity and pain over the duration of the intervention. Greater provider attestation to the use of the CDS tool appeared to be associated with larger decreases in disease activity among black children compared to white children, and therefore the effects of CDS on treatment preferences and provider-patient interactions by race will warrant further exploration in a larger study of underserved minorities.

Similar rates of improvement over the duration of this study suggest that the intervention as a whole was equally effective across different racial groups. In the previous description of the TTT-CDS intervention, there was an increase in the use of biologic therapy after implementation that may have contributed to the improvements in disease activity over time [17]. Notably, increases in biologic use over time did not differ by race in this analysis, though there may have been small differences that we were unable to detect due to the relatively small number of black children. As there were several components to the intervention, including systematic data collection, a patient registry, automated provider performance feedback, treatment algorithms, and target attestation, additional research is needed to determine which aspect of this multicomponent intervention has the greatest potential impact on disease outcomes.

Despite similar improvements across all racial groups, we observed that black children with polyarticular JIA had persistently greater disease activity as assessed by both the treating physician and patient/ caregiver. Our findings parallel previous studies demonstrating that black children in the US with polyarticular JIA have greater joint damage [3]. Similarly, several observational studies in adults with rheumatoid arthritis have demonstrated significant racial and ethnic disparities in various patient outcomes [4, 23, 24]. In a longitudinal assessment of the Consortium of Rheumatology Researchers of North America (CORRONA) registry, black patients with rheumatoid arthritis had consistently greater disease activity levels, lower rates of clinical remission, and worse functional status compared to their white counterparts over a 5 year period, despite similar improvements across both groups [23]. The etiology of racial disparities in health outcomes are multifactorial, and involve a combination of socioeconomic factors, cultural preferences, genetic factors, unconscious bias, physician trust and attitudes toward treatment, as well as patient-physician communication [9]. There is data supporting the hypothesis that a “window of opportunity” exists for arthritis, in which earlier initiation of biologic therapy is associated with better outcomes over time [25]. As a result, access to health care and acceptability of treatment may play especially important roles in arthritis outcomes. In our study, insurance type as a marker of access did not explain the observed racial differences in health outcomes. While we cannot minimize the importance of macrolevel inequities and other unmeasured socioeconomic determinants, our findings highlight the need to identify other potentially modifiable sources of disparity at the patient-provider level.

This study explores whether standardization of care using a health systems approach has the potential to improve racial disparities in JIA outcomes by modifying provider-patient level decision-making and reducing treatment variability. Two studies from a single U.S. cohort of adults with rheumatoid arthritis have reported that black patients were, on average, more risk averse and less likely to prefer aggressive treatment than white patients [26, 27]. However, individual preferences may vary, and what on the surface appears to be a preference may actually be rooted in the historical context of generations of institutionalized racism in the U. S and resulting mistrust of the health care system [28]. To date, there are no studies examining racial differences in caregiver preferences for treatment of children with JIA. Moreover, there is a paucity of literature on the effect of interventions on changing patient preferences, either by addressing disparities in knowledge/expectations about a treatment or improving trust in providers [28]. In this intervention, the decision support algorithms and individual disease target attestations could be reviewed with families at the point of care, which has the potential to influence physician trust, communication, and attitudes toward treatment. An interesting, albeit exploratory, finding from our study was the greater effect of provider attestation to CDS use on disease activity among black children with prevalent JIA compared to white children. This will need to be reproduced in a larger study, as our small sample size of black patients precludes drawing firm conclusions about the role of CDS. While our intervention was not specifically designed to address racial differences in treatment preference, we hypothesize that by systematically affecting provider-level and patient-level interactions, the intervention may have had the unintended consequence of improving patient-physician communication about treatment. Larger studies with a qualitative component are needed to understand treatment preferences and the effect of TTT and CDS to identify strategies to modify racial disparities at the level of provider-patient interactions.

There are several limitations to this study. The number of black children in our cohort was small, and therefore our results need to be interpreted with caution. There may have also been insufficient power to detect small differences by race toward the end of the observation period. There were also too few Hispanic patients to analyze meaningful differences by ethnicity. Moreover, due to unequally spaced visits as this was not a protocolized study, we were unable to directly compare mean disease activity level at fixed intervals across race categories. Lastly, as all patients were exposed to the intervention and disease activity scores were not assessed systematically prior to implementation, we are unable to determine whether there would have been similar improvements by race in the absence of the intervention.

## Conclusion

In conclusion, a TTT-CDS intervention designed to improve disease activity outcomes among a U.S. cohort of children with polyarticular JIA resulted in individual level and population level decreases in disease activity scores across all racial groups. Despite similar rates of improvement, racial differences in JIA outcomes persisted over time. More work is needed to address the underlying etiologies of racial disparities in JIA disease outcomes at the level of the health system and patient-provider interactions. Efforts to improve uptake of CDS and systematically influence provide-patient communication about treatment decisions deserve further exploration as a potential strategy to reduce disease activity among black children and other underserved minorities with JIA. Future prospective studies are also needed to determine whether broader implementation of approaches to standardize disease monitoring and treatment can reduce racial health care disparities across real-world clinical settings.

## Data Availability

The datasets analyzed in this study are not publicly available due to information that could compromise patient privacy, but a limited dataset is available from the corresponding author on reasonable request and with permission of the Children’s Hospital of Philadelphia.

## Abbreviations

CDS: Clinical decision support
cJADAS-10: Clinical juvenile arthritis disease activity score
DMARD: Disease-modifying antirheumatic drug
ILAR: International League Against Rheumatism
IQR: Interquartile range
JIA: Juvenile idiopathic arthritis
PROMIS: Patient Reported Outcomes Measurement Information System®
RF: Rheumatoid factor
TTT: Treat-to-target
